# Parafoveal Dark Adaptation in Early and Intermediate Age-Related Macular Degeneration

**DOI:** 10.1167/iovs.67.5.74

**Published:** 2026-05-29

**Authors:** Georg Ansari, Jeannine Oertli, Laura Mächler, Theresa Lipsky, Brett G. Jeffrey, Catherine A. Cukras, Nicolas Feltgen, Caroline C. W. Klaver, Kristina Pfau, Maximilian Pfau

**Affiliations:** 1 Department of Ophthalmology, University Hospital Basel, Basel, Switzerland; 2 National Eye Institute, National Institutes of Health, Bethesda, Maryland, United States; 3 Institute of Molecular and Clinical Ophthalmology Basel (IOB), Basel, Switzerland; 4 Department of Ophthalmology, Erasmus University Medical Center, Rotterdam, The Netherlands; 5 Department of Ophthalmology, University of Bonn, Bonn, Germany

**Keywords:** fundus-controlled dark adaptometry, dark adaptation, early age-related macular degeneration (AMD), intermediate AMD, biomarker, functional endpoints

## Abstract

**Purpose:**

Rod-mediated dark adaptation delays are among the earliest functional abnormalities in age-related macular degeneration (AMD), preceding photoreceptor loss. This study evaluated whether parafoveal fundus-tracked dark adaptometry at 2 degrees detects earlier rod dysfunction than mid-macular loci (4 degrees and 6 degrees) and assessed the diagnostic performance of dynamic and steady-state parameters across eccentricities.

**Methods:**

In this cross-sectional study, 35 patients with predominantly early/intermediate AMD and 35 healthy volunteers across a broad range of ages underwent fundus-controlled dark adaptometry (S-MAIA-2; iCare/CenterVue) and multimodal imaging. After standardized bleaching, Goldmann III sized (0.43 degrees) cyan stimuli were presented 2 degrees, 4 degrees, and 6 degrees temporal to the fovea. Dark-adaptation curves were modeled to derive rod intercept time (RIT), final (rod) threshold (FT), and cone threshold (CT), each compared with normative data. Diagnostic accuracy was quantified using covariate-adjusted receiver operating characteristic (ROC) analyses, to account for age.

**Results:**

Among 70 analyzed eyes, RIT was abnormal in 86% of AMD eyes at 2 degrees, 69% at 4 degrees, and 60% at 6 degrees, whereas FT and CT were less frequently abnormal (29% to 51% and 17% to 26%, respectively). Median RIT at 2 degrees reached 60 minutes in the AMD cohort, indicating absence of rod function within the test duration in many eyes. RIT achieved the highest diagnostic accuracy, with covariate-adjusted area under the curve (AUC) values of 0.91 (95% credible intervals [CrI] = 0.80–0.97) at 2 degrees, 0.88 (95% CrI = 0.77–0.96) at 4 degrees, and 0.87 (95% CrI = 0.76–0.95) at 6 degrees.

**Conclusions:**

Fundus-tracked dark adaptometry enables spatially precise assessment of parafoveal rod recovery. Parafoveal RIT prolongation represents the earliest and most frequent functional abnormality in AMD and demonstrates excellent diagnostic performance, supporting its potential as a sensitive functional biomarker for early disease and therapeutic trials.

Age-related macular degeneration (AMD) is the leading cause of legal blindness in developed countries, with limited therapeutic options currently available.^1^^,^^2^ Globally, the prevalence of any form of AMD has been estimated at 196 million, and it is projected to rise to 288 million in 2040.^2^

AMD initially affects Bruch’s membrane and the choriocapillaris, resulting in a characteristic appearance resembling an “oil spill” in histopathology.^3^^,^^4^ With time, these deposits coalesce to form drusen between Bruch's membrane inner collagenous layer and the basal lamina of the retinal pigment epithelium (RPE).^3^^,^^4^ Enlargement of such drusen together with pigmentary abnormalities indicate an increased likelihood of progression toward late-stage disease.^5^^–^^10^ Late-stage disease is characterized by two major trajectories: either geographic atrophy (GA), marked by the loss of RPE and overlying photoreceptors, or neovascular AMD, characterized by angiogenesis and exudation, both of which culminate in central vision loss.

Microstructural investigations – histopathology and in vivo imaging studies – converge on a parafoveal annulus, extending between eccentricities of approximately 750 µm (2.6 degrees) and 1250 µm (4.3 degrees), as the region of greatest susceptibility for the incidence of GA.^11^^,^^12^ This ring also corresponds closely to the broader parafoveal ring spanning 0.5 to 3 mm eccentricity, where rod photoreceptor density undergoes its steepest decline with aging and AMD.^13^

Psychophysical studies likewise localize early rod dysfunction to the parafovea, where rod-mediated vision is disproportionately vulnerable to aging and AMD.^14^^–^^19^ As early as 1993, Steinmetz and coworkers reported delayed rod-mediated dark adaptation in patients with AMD, that is, dynamic dysfunction of the rod system occurring even when steady-state thresholds remained normal.^16^ Further, a gradient of increasing impairment was postulated toward the fovea (closest test locus at 3 degrees eccentricity) in the discussion section, but no quantitative analysis was performed.^16^ Subsequent investigations confirmed this pattern using dark adaptometry at multiple predefined retinal locations (testing as close as 4 degrees eccentricity),^20^^–^^22^ or repeated unifocal dark adaptometry (comparing 5 degrees and 12 degrees),^23^ or using arc shaped stimuli at 3 degrees and 5.5 degrees.^24^^,^^25^ However, quantitative data characterizing dark-adaptation delays in the actual region of interest – the parafoveal “high-risk” annulus – remain absent to date.

Recently, the advent of fundus-tracked dark adaptometry has enabled localized assessment of rod-mediated vision within the parafovea. Active fundus tracking reduces reliance on precise fixation by participants, allowing the use of dim fixation lights that minimize interference with dark adaptation. Furthermore, using small stimuli facilitates spatially specific testing (i.e. Goldmann III [0.43 degrees diameter, equivalent to 125 µm] stimuli instead of Goldmann V [1.72 degrees] or 2 degrees stimuli). Together, these advances provide the capability to probe rod function specifically within the parafoveal annulus, identified microstructurally as the earliest region of susceptibility in AMD.

As the logical next step, we evaluated the feasibility of fundus-tracked dark adaptometry in AMD, compared outcomes with controls, and assessed its diagnostic validity across dark-adaptation parameters and stimulus eccentricities. To establish concurrent validity, we performed structure–function analyses with established structural biomarkers of AMD severity. Finally, we examined the inter-relationships among dark-adaptation parameters to explore whether the nonlinear associations of functional loss are indicative of a functional disease sequence.

## Methods

### Study Design

In this prospective study, healthy volunteers and patients with AMD were recruited and examined in a tertiary referral center (University Hospital Basel). The study was approved by the ethics committee (BASEC ID: 2022-01243 [https://raps.swissethics.ch/]) and conducted in accordance with the Declaration of Helsinki. All participants were informed of the study's nature and provided written informed consent before participating in study-related examinations.

Healthy volunteers were selected on the basis of a normal ophthalmic examination and unremarkable optical coherence tomography (OCT) imaging. Exclusion criteria included the presence of any structural retinal abnormality on OCT or a history of ocular disease. Healthy volunteers were enrolled over a broad range of ages.

Participants included eyes with early or intermediate AMD (per eye Beckman classification) and a small subset with GA.^26^ GA eyes were required to have an intact retina at the test locations (2 degrees, 4 degrees, and 6 degrees temporal to the fovea) to allow meaningful measurement of dark adaptation. GA eyes were included to extend the functional spectrum of disease severity.

Other inclusion criteria for patients with AMD encompassed visual acuity of <1.0 logMAR, clear optic media, and no treatment with anti-VEGF in study eye. Exclusion criteria were the inability to provide informed consent, severe claustrophobia, previous ocular surgeries (other than cataract surgery, YAG laser capsulotomy, and refractive laser surgery), and concurrent ophthalmic conditions in the study eye that might independently affect visual acuity, as judged by the investigator. Of note, visual acuity inclusion criteria were intentionally set at a permissive threshold (logMAR <1.0) to avoid any exclusion of patients based on visual function alone. In practice, participants in both the control and AMD groups demonstrated good best-corrected visual acuity (BCVA) and stable fixation.

The study eye was selected according to a predefined procedure. Eyes with no history of exudative macular neovascularization (i.e. without previous anti-VEGF treatment) were chosen. If both eyes met this criterion, the eye with the superior BCVA was designated as the study eye. In cases where BCVA was identical in both eyes, a random eye was selected.

### Imaging: Spectral-Domain Optical Coherence Tomography, Fundus-Autofluorescence and Color Fundus Photography

Spectral domain OCT (SD-OCT) imaging of the macula was obtained with a Heidelberg Spectralis device (Heidelberg Engineering, Heidelberg, Germany) with a 30 degrees × 25 degrees (121 B-scans, HR mode, enhanced Automatic Real Time-Function [ART] averaged 25 scans). Short-wavelength fundus autofluorescence (FAF) images (30 degrees × 30 degrees, HS mode, and ART 20) were performed using the same device. True-color color fundus photography (CFP) was obtained with the Zeiss Clarus 500 device (Carl Zeiss AG, Jena, Germany).

### Fundus-Controlled Dark-Adaptometry

**Figure 1.**
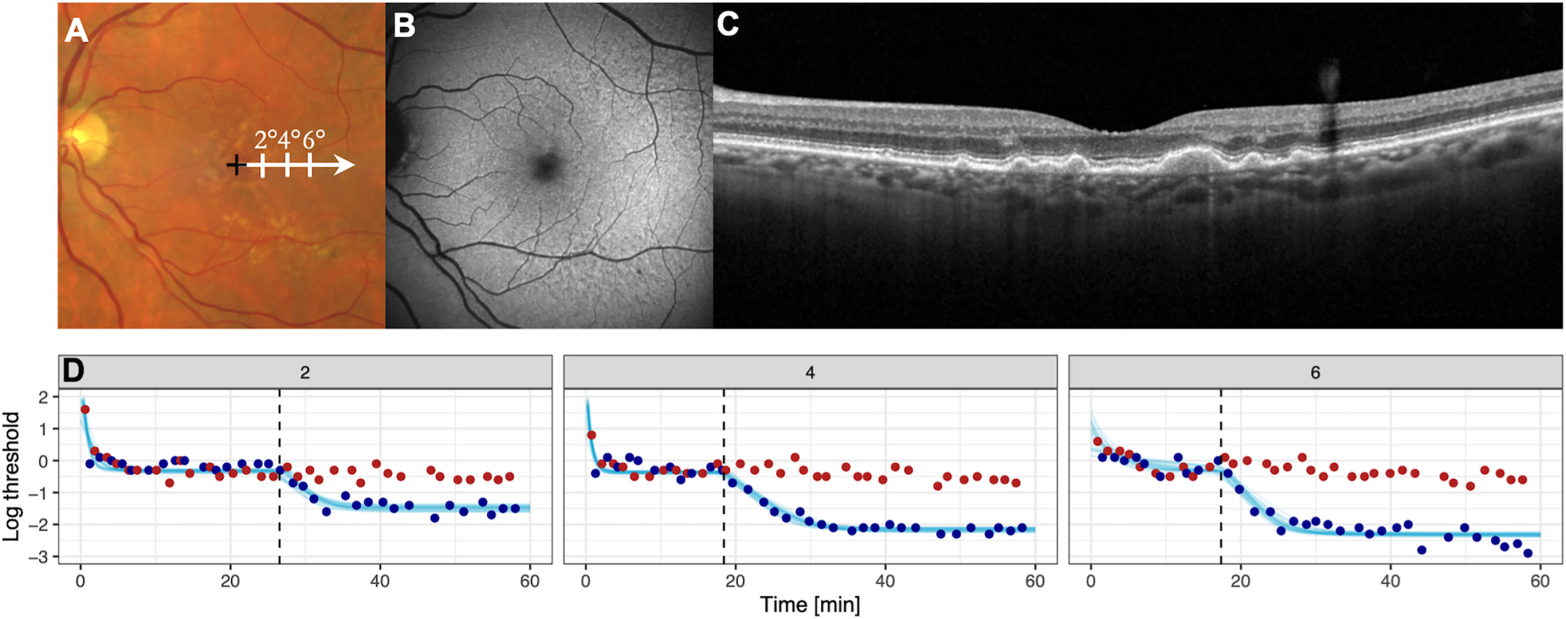
**Fundus-controlled dark adaptometry in age-related macular degeneration (AMD).** The panels **(A–C**) show multimodal imaging of a representative patient with color fundus photography **A**, fundus autofluorescence **B**, and spectral-domain optical coherence tomography **C** imaging. Panel **A** illustrates the stimulus locations along the horizontal meridian at 2 degrees, 4 degrees, and 6 degrees temporal to the fovea (indicated by the *black cross*). Panel (**D**) displays the corresponding dark-adaptation curves for red (*red dots*) and cyan (*blue dots*) stimuli at eccentricities of 2 degrees, 4 degrees, and 6 degrees. The y-axis indicates retinal sensitivity (log threshold), and the x-axis shows time (minutes) after the initial bleaching. The *sky-blue lines* represent fitted model curves; the *vertical dashed line* denotes the cone–rod break. The foveal location was estimated visually in the color fundus photography for illustrative purposes only and was not involved in any of the quantitative analyses.

Patients underwent fundus-controlled dark adaptometry using the S-MAIA-2 device (iCare/CenterVue, Padova, Italy). Patients underwent 45 minutes of dark adaptation (healthy volunteers 30 minutes) and, in this time, pupils were dilated at least 3 times with mydriatic eye drops (1% phenylephrine with 0.5% tropicamide). The protocol used a full-field bleach (634 photopic cd/m², equivalent to 946 scotopic cd/m²) lasting 5 minutes (equivalent to a 59% rhodopsin bleaching, or a 54% cone pigment bleach, see appendix in Flynn et al. for calculation)^21^ from the MonCvONE device (Metrovision, Perenchies, France). Immediately after bleaching, patients were moved to the S-MAIA-2 device. Cyan stimuli (peak wavelength of 505 nm) and red stimuli (627 nm) were used. Both stimuli had a duration of 200 ms, a Goldmann III stimulus size of 0.43 degrees (equivalent to 125 µm), and a dynamic range of 0 to 36 decibel (dB).^27^ Red and cyan stimuli were presented interleaved at retinal loci 2 degrees, 4 degrees, and 6 degrees temporal to fixation (Fig. 1), in ascending order (i.e. from most central to most peripheral locus). Patients were tested continuously in a 5 up 1 down strategy, that is, the stimulus was presented 5 dB darker after a response and 1 dB brighter if no response was recorded. After one response, thresholds were tested on the next location. If subjects reported fatigue during the test, short breaks were made for 1 minute during the tests to avoid prolonged recording breaks. Testing continued for up to 60 minutes or was terminated earlier if steady-state rod thresholds were achieved at all loci.

Dark adaptation curve estimations were modeled using a Bayesian nonlinear regression with the R package *brms*.^28^ The curves were fitted using a biphasic model defined as follows:

$$\begin{array}{ccc} threshold & = & c_{t}+\left(t_{0}-c_{t}\right)*\;e^{-\frac{t}{\;\tau }}\\ & & +log_{10}\left(10^{S_{2}*\left(t-CRB\right)}+\;10^{t_{f}-c_{t}}\right) \end{array}$$

if time > CRB, otherwise:

$$threshold=c_{t}+\left(t_{0}-c_{t}\right)*\;e^{-\frac{t}{\;\tau }}$$

with the parameters: *c_t_* = cone threshold, *t*_0_ = initial threshold at time 0, τ = exponential time constant, *S*_2_ = rod adaptation slope, *CRB* = cone-rod break, and *t_f_* = final (rod) threshold, all estimated as nonlinear terms (Supplementary Fig. S1). Model fitting was performed using weakly informative priors that encompassed the full range of physiologically plausible values for each curve parameter (Supplementary Table S1). These priors were intentionally broad and applied uniformly to all dark-adaptation curves so as to constrain the solutions to realistic limits without exerting meaningful influence on the estimated fits. The same prior structure was used for every participant to maintain analytic consistency.

Using nonlinear curve fitting, we determined cone thresholds (CTs) and final (rod) thresholds (FTs) in logUnits.^28^ We chose −1.4 logUnits as the criterion threshold for RIT because it lies approximately 1 logUnit below the normal CT. The researcher manually reviewed all curves. If there was no evidence of a cone-rod break (i.e. no separation between cyan and red stimuli) within the 60-minute test, the FT was considered cone-mediated, and RIT was recorded as 60 minutes.

### Statistical Analysis

Statistical analyses were performed in *R* using the add-on packages *tidyverse*,^29^ *ggplot2*,^30^ *table1*,^31^ *tidymodels*,^32^ *dplyr*,^33^ and *caret*.^34^

To estimate normal limits from the healthy volunteer data, we applied Bayesian linear mixed-effects regression models to estimate the relationship between a given dependent variable (CT, FT, and RIT) and age and retinal eccentricity. Age was modeled as a fixed effect, retinal position as a fixed categorical effect, and subject-specific variability was accounted for by including a random intercept for each participant. Weak priors were specified based on previously published normative datasets.^35^^,^^36^ The predicted values were summarized by a median and 95% prediction interval.

To evaluate the diagnostic accuracy of CT, FT, and RIT for distinguishing between healthy individuals and patients with AMD at different eccentricities, covariate-adjusted receiver operating characteristic (ROC) curves with age as a covariate were generated using the R package *ROCnReg.*^37^ To account for inter-group age differences, the diagnostic accuracy estimates were covariately adjusted for age (i.e. yielding a more conservative estimate than “pooled” ROC analyses).

To explore inter-relationships among dark-adaptation parameters, the parameters were expressed relative to normative limits: that is, RIT in terms of RIT delay and CT, and FT in terms of threshold deviation. Scatter plots were generated to visualize nonlinear dependencies and to identify potential thresholds beyond which steady-state sensitivity declined.

For structure-function analysis, we evaluated the relationship between RIT and established biomarkers of AMD severity (AMD severity of the study eye and fellow eye [early, intermediate, and late - according to the Beckman classification], presence/absence of subretinal drusenoid deposits (SDDs) in the study eye, and age) using linear regression analysis.

## Results

### Cohort Characteristics

Thirty-five patients with clinically confirmed AMD in both eyes, with a median age of 70.9 years (interquartile range [IQR] = 65.5–79.3 and overall range = 47.3–86.2), and 35 healthy volunteers (54.8 years [IQR = 28.1– 64.4 and overall range = 21.7–82.0]) were included in this study (Table 1). Median visual acuity in the study eye was −0.07 logMAR (IQR = −0.07 to 0.11) in the AMD cohort and −0.07 logMAR (IQR = −0.07 to −0.04) in healthy individuals. SDDs were present in 10 (28.6%) patients with AMD (Supplementary Tables S2, S3).

**Table 1.** Cohort Characteristics

	AMD (*n* = 35)	Healthy Volunteers (*n* = 35)
Age, y		
Median [IQR]	70.9 [65.5 to 79.3]	54.8 [28.1 to 64.4]
Range	47.3 to 86.2	21.7 to 82.0
Sex		
F	22 (62.9%)	21 (60.0%)
M	13 (37.1%)	14 (40.0%)
Study eye		
Left	14 (40.0%)	21 (60.0%)
Right	21 (60.0%)	14 (40.0%)
Subretinal drusenoid deposits		
Absent	25 (71.4%)	NA
Present	10 (28.6%)	NA
Study eye visual acuity [logMAR]		
Median [IQR]	−0.07 [−0.07 to 0.11]	−0.07 [−0.07 to −0.04]
Diagnosis: Study eye		
eAMD	11 (31.4%)	0 (0%)
iAMD	20 (57.1%)	0 (0%)
Late AMD: GA	4 (11.4%)	0 (0%)
None	0 (0%)	35 (100%)
Diagnosis: Fellow eye		
eAMD	10 (28.6%)	0 (0%)
iAMD	17 (48.6%)	0 (0%)
Late AMD: GA	6 (17.1%)	0 (0%)
Late AMD: nAMD	2 (5.7%)	0 (0%)
None	0 (0%)	35 (100%)

eAMD, early AMD; iAMD, intermediate AMD; nAMD, neovascular AMD; GA, geographic atrophy.

### Dark Adaptation in the Parafovea (2 Degrees) Versus 4 Degrees and 6 Degrees Eccentricity

**Figure 2.**
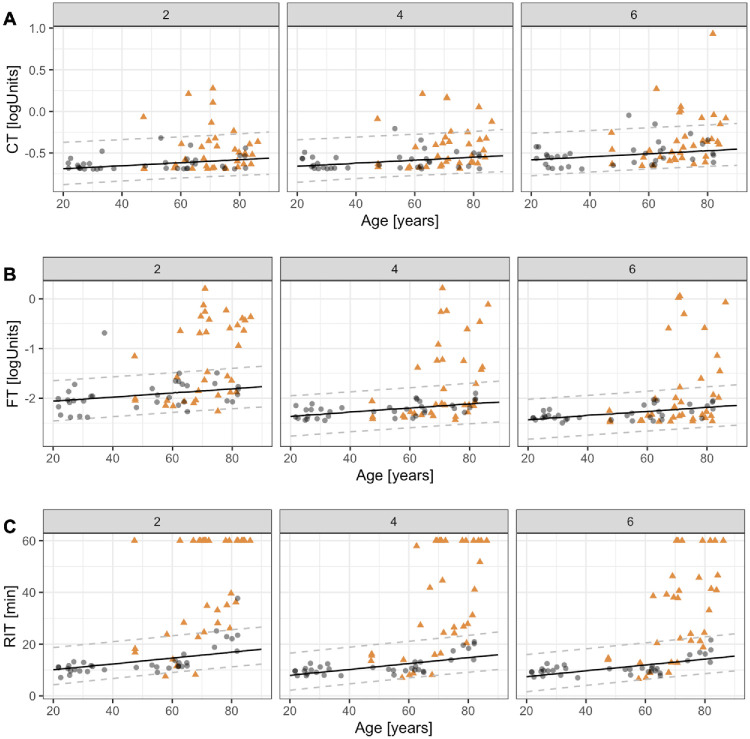
**Dark adaptometry outcomes in age-related macular degeneration (AMD) and healthy controls.** Cone threshold (CT, row **A**), final (rod) threshold (FT, row **B**), and rod intercept time (RIT, row **C**) are plotted against age for test loci at 2 degrees, 4 degrees, and 6 degrees eccentricity. *Gray circles* represent healthy controls, and *orange triangles* represent patients with AMD. *Solid lines* indicate the estimated mean random-slope model for healthy participants, with *dashed lines* showing the corresponding 95% prediction intervals.

Cone-mediated thresholds were largely preserved across eccentricities. Median (IQR) steady-state cone thresholds were −0.49 logUnits (IQR = −0.63 to −0.32) in the AMD cohort, corresponding to a median CT deviation from normative predictions of −0.05 logUnits (IQR = −0.21 to 0.06) at 2 degrees, −0.03 logUnits (IQR = −0.24 to 0.07) at 4 degrees, and −0.07 logUnits (IQR = −0.30 to 0.07) at 6 degrees. Considering the 95% prediction intervals derived from healthy volunteers, CTs fell outside the normal limits in only a minority of AMD eyes: 6 (17%) at 2 degrees, 7 (20%) at 4 degrees, and 9 (26%) at 6 degrees (Fig. 2).

Rod mediated steady-state thresholds were −1.99 logUnits (IQR = −2.29 to −0.69) in patients with AMD, corresponding to a median rod threshold deviation from normative predictions of −0.37 logUnits (IQR = −1.32 to 0.09) at 2 degrees, −0.04 logUnits (IQR = −0.89 to 0.13) at 4 degrees, and 0.07 logUnits (IQR = −0.51 to 0.17) at 6 degrees. In contrast to CTs, rod-mediated steady-state thresholds were more frequently abnormal, exceeding limits in 18 (51%) eyes at 2 degrees, 13 (37%) at 4 degrees, and 10 (29%) at 6 degrees temporal to the fovea.

The most striking dysfunction, however, emerged in the kinetics of rod recovery. RITs were markedly prolonged in AMD, most severe at 2 degrees eccentricity, with a median (IQR) of 60 minutes (IQR = 25.4–60.0), indicating absence of rod function within the test duration in many eyes. RIT declined with increasing distance to the fovea, with medians of 31.2 minutes (IQR = 16.3–60.0) at 4 degrees and 33.1 minutes (IQR = 14.4–46.0) at 6 degrees.

When compared with normative predictions, RIT was prolonged by a median (IQR) of 42.38 minutes (IQR = 9.27–44.07) at 2 degrees, 16.23 minutes (IQR = 4.80–45.06) at 4 degrees, and 18.71 minutes (IQR = 3.86–31.82) at 6 degrees. RIT fell outside normal limits in 30 (86%) eyes at 2 degrees, 24 (69%) at 4 degrees, and 21 (60%) at 6 degrees (Supplementary Table S4).

### Discriminating Accuracy Between AMD and Healthy Controls

**Figure 3.**
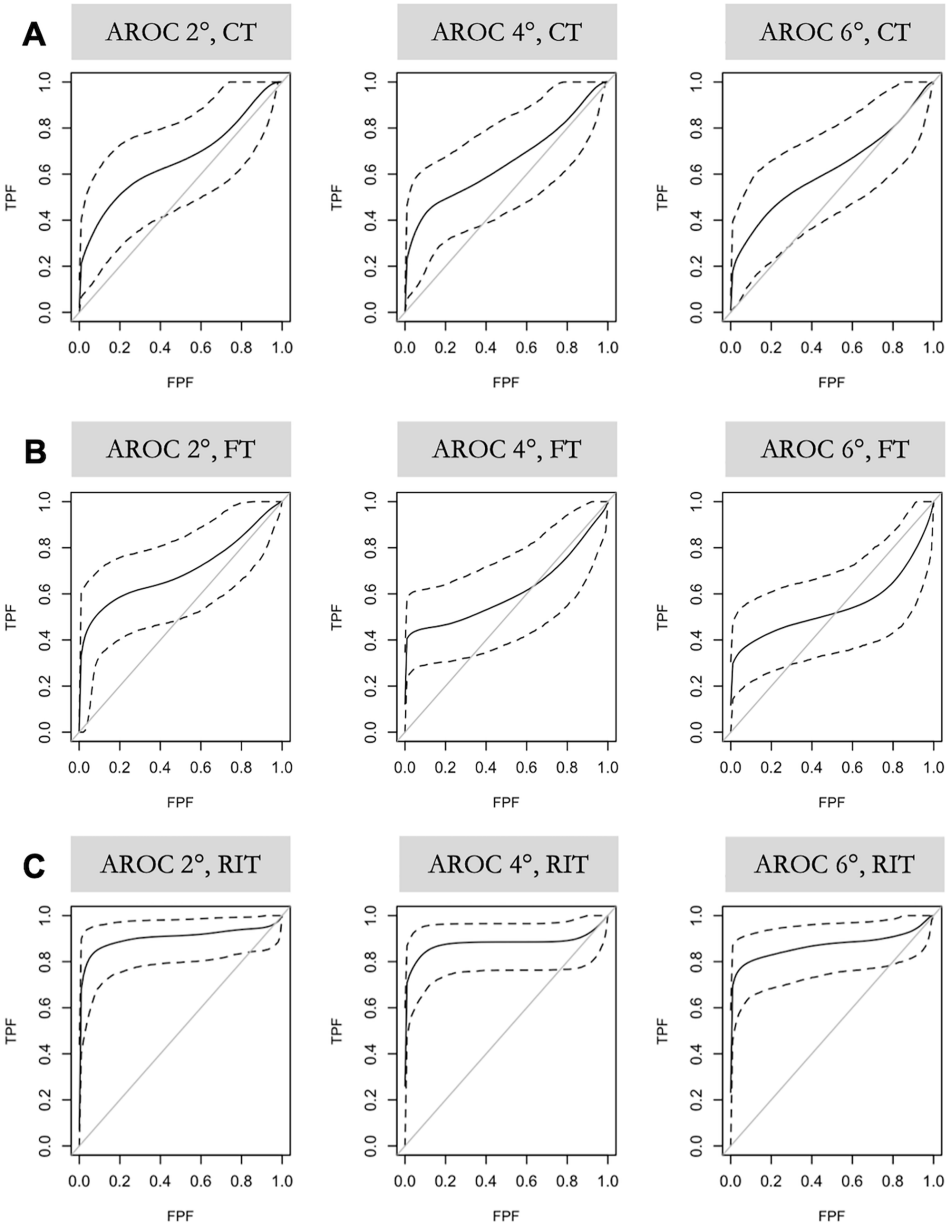
**Covariate-adjusted receiver operating characteristic (ROC) curves for differentiating AMD from healthy controls.** ROC curves are shown for cone threshold (CT, row **A**), final rod threshold (FT, row **B**), and rod intercept time (RIT, row **C**) (*rows*) across test eccentricities of 2 degrees, 4 degrees, and 6 degrees (*columns*). Each panel plots the true positive fraction (TPF) against the false positive fraction (FPF). *Solid lines* represent mean ROC curves, and *dashed lines* indicate the corresponding 95% credible intervals.

ROC analyses adjusted for age demonstrated that RIT most effectively distinguished eyes with AMD from healthy controls at all tested eccentricities (Fig. 3). Discriminative performance was highest at 2 degrees, with a covariate-adjusted ROC area under the curve (ROC-AUC) of 0.91 (95% CrI = 0.80–0.97), and remained robust at 4 degrees (0.88, CrI = 0.77–0.96) and 6 degrees (0.87, CrI = 0.76–0.95).

At every test location, the dynamic measure of rod recovery (RIT) surpassed steady-state thresholds in separating disease from normal. Final rod threshold yielded lower ROC-AUCs (0.70, CrI = 0.56–0.83, 0.61, CrI = 0.46–0.75, and 0.55, CrI = 0.40–0.69), and CTs performed only modestly as well, 0.66 (CrI = 0.51–0.80), 0.65 (CrI = 0.50–0.79), and 0.62 (CrI = 0.47–0.76).

### Inter-relationships Among Dark Adaptation Parameters

**Figure 4.**
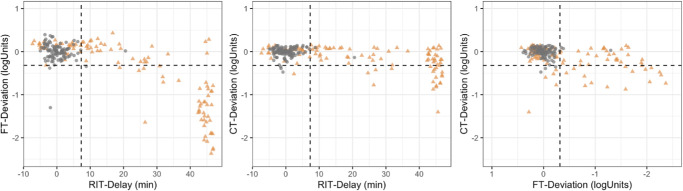
**Pairwise associations between dark adaptometry outcomes in patients with age-related macular degeneration and healthy controls.** The *left panel* shows the relationship between rod-intercept time (RIT) delay and final rod threshold (FT) deviation. The *middle panel* illustrates the association between RIT delay and cone threshold (CT) deviation. The *right panel* depicts the relationship between FT deviation and CT deviation. The *grey circles* represent healthy controls, whereas the *orange triangles* represent patients with AMD. *Dashed lines* indicate the estimated 97.5th percentile (upper quantile) of the healthy cohort for RIT delay, FT deviation, and CT deviation.

The relationships between dynamic and steady-state measures of dark adaptation were nonlinear (Figs. 4A, 4B). When RIT delays were modest (<30 minutes), FTs were generally preserved, with FT deviations clustering near normal limits. Except for two outlier loci, no substantial FT deviations occurred within this range. In contrast, if RIT delays reached approximately 40 minutes, FT deviations were severe, indicating an absence of measurable rod function over the whole test duration.

A similar but less pronounced trend was observed for CT. The CT deviations were observable at locations with RIT delays of 40 minutes or longer, but with a smaller magnitude compared to the FT deviations. The relationship between CT deviations and FT deviations was linear, indicating that steady-state deficits in the two systems occur concurrently. However, the magnitude of impairment differed substantially: FT deviations reached as much as −2 log units, whereas CT deviations were generally limited to approximately −1 log unit.

### Structure-Function Associations

Multivariate regression (Table 2) identified subretinal drusenoid deposits as the strongest determinant of delayed rod recovery, with RIT prolongations of +18.47 minutes at 2 degrees, +24.36 minutes at 4 degrees, and +26.31 minutes at 6 degrees (all *P* < 0.001). Intermediate and late AMD were independently associated with additional RIT delays of 20 to 33 minutes across all eccentricities (all *P* < 0.001), whereas early AMD exerted no significant effect. In our model, age modestly increased RIT (+1.62 minutes/decade at 6 degrees to +2.41 minutes/decade at 2 degrees). Fellow-eye diagnosis showed no consistent association with RIT across eccentricities. The models accounted for 71% to 79% of total RIT variance, underscoring the strong, coherent relationship between retinal structure and rod-mediated function.

**Table 2.** Association Between Rod Intercept Time (RIT) and Subretinal Drusenoid Deposits (SDD) as Well as AMD-Severity in the Study Eye (SE) and the Fellow-Eye (FE), With Adjustment for Age

	RIT 2 Degrees [min]			RIT 4 Degrees [min]			RIT 6 Degrees [min]		
Predictors	Estimates	CI	*P* Value	Estimates	CI	*P* Value	Estimates	CI	*P* Value
(Intercept)	1.93	−7.32 to 11.18	0.678	1.98	−6.64 to 10.59	0.648	3.10	−3.33 to 9.54	0.339
SDD	18.47	8.79 to 28.15	**<0.001**	24.36	15.34 to 33.37	**<0.001**	26.31	19.57 to 33.04	**<0.001**
Diagnosis SE (eAMD)	5.07	−29.85 to 39.99	0.773	−0.79	−33.32 to 31.74	0.961	−6.31	−30.60 to 17.99	0.605
Diagnosis SE (iAMD)	32.62	17.99 to 47.24	**<0.001**	27.86	14.24 to 41.48	**<0.001**	24.47	14.30 to 34.64	**<0.001**
Diagnosis SE (late AMD)	28.87	13.78 to 43.96	**<0.001**	28.01	13.96 to 42.07	**<0.001**	20.59	10.09 to 31.09	**<0.001**
Diagnosis FE (eAMD)	4.14	−30.88 to 39.16	0.814	8.46	−24.16 to 41.09	0.606	10.47	−13.90 to 34.84	0.394
Diagnosis FE (iAMD)	−12.33	−26.44 to 1.79	0.086	−13.92	−27.07 to −0.77	**0.038**	−14.25	−24.07 to −4.42	**0.005**
Diagnosis FE (lateAMD)	−8.57	−34.51 to 17.37	0.511	−10.09	−34.25 to 14.08	0.407	−2.99	−21.04 to 15.05	0.741
Age [decade]	2.41	0.68 to 4.14	**0.007**	1.97	0.36 to 3.58	**0.017**	1.62	0.42 to 2.83	**0.009**
Observations	70			70			70		
*R* ^2^/*R*^2^ adjusted	0.737/0.702			0.747/0.714			0.817/0.793		

CI, 95% confidence interval.

The *P* values in bold represent statistical significance.

## Discussion

We found that fundus-tracked dark adaptometry using small, spatially precise stimuli to quantify rod-recovery kinetics in the parafovea more reliably distinguishes controls from patients with AMD than steady-state threshold measurements. Moreover, the diagnostic accuracy was best in the parafovea. The results indicate that a mechanism-linked, spatially appropriate functional biomarker can enhance early disease staging and may help guide interventions aimed at modifying the degenerative trajectory before atrophy develops.

We used fundus-controlled testing at 2 degrees, 4 degrees, and 6 degrees eccentricities, modeling full dark-adaptation curves to derive RIT, FT, and CT, which revealed that RIT abnormalities were nearly ubiquitous at 2 degrees (86%), declining gradually at 4 degrees (69%) and 6 degrees (60%). FT abnormalities were less common (51% at 2 degrees and 29% at 6 degrees), and CT abnormalities were rare (17% to 26% across loci). Median RIT at 2 degrees was 60 minutes, but less delayed at 4 degrees and 6 degrees. Jointly, these data establish a clear spatial gradient, with the parafoveal locations showing the earliest and most frequent dysfunction. Prior work had hinted that dark adaptation deficits increase toward the fovea, but earlier free-viewing perimetry devices could not test closer than 3 degrees,^16^ 4 degrees,^21^^,^^22^^,^^38^ or even farther eccentric.^23^ The recent development of fundus-tracked dark adaptometry with Goldmann III sized stimuli^39^ now enabled us to test patients in a spatially specific manner and confirm that diagnostic accuracy peaks parafoveally (2 degrees). This locus is consistent with the spatial annulus of peak rod photoreceptor loss in histopathology,^13^ as well as retinal thinning observed in healthy eyes with a genetic susceptibility to AMD in the UK Biobank.^40^

Dynamic dysfunction (in terms of RIT delay) consistently outperformed steady-state thresholds in differentiating AMD from controls. Notably, the relationship between RIT delay and FT was nonlinear: typically, steady-state rod dysfunction was mostly confined to locations with RIT delays of >40 minutes. A similar trend was seen for the relationship between RIT and CTs. This supports the longstanding hypothesis that dynamic dysfunction precedes steady-state dysfunction in AMD. However, most earlier studies were limited by testing protocols that terminated before FTs were measurable and did not provide confirmatory data. Flynn et al. showed that dark-adaptation responses can be categorized into distinct phenotypes—normal, isolated dynamic delay, and combined dynamic and steady-state impairment—but did not establish a quantitative RIT threshold at which steady-state dysfunction first becomes evident.^21^ The longitudinal National Institutes of Health (NIH) dark adaptation study reported an RIT >22.8 minutes (i.e. RIT delay of 11 minutes with their settings) for the onset of CT elevation,^41^ which differs considerably from our data. This is likely attributable to the unifocal testing at 5 degrees eccentricity (1.7 degrees diameter stimulus), where RIT delays tend to be less pronounced compared to 2 degrees. Mechanistically similar relationships have been noted in other Bruch's membrane diseases: L-ORD,^42^ Sorsby fundus dystrophy,^43^^,^^44^ and Pseudoxanthoma elasticum,^45^ in which dynamic dysfunction may occur in isolation and steady-state loss only later, when metabolic disruption of the RPE–photoreceptor interface becomes irreversible.

The 5 minute full-field bleach, corresponding to approximately 59% rhodopsin bleaching, was intentionally set at a relatively high level to enable the detection of subtle functional effects. This value was chosen slightly below previously reported bleaching levels (76% and 82%).^46^^,^^47^ Given the robustness of our findings, future studies using fundus-controlled dark adaptometry may achieve comparable discriminating accuracy between early AMD stages even with lower bleaching intensities, similar to the 30% bleach that has been established previously.^21^^,^^48^ Given our observed median RIT of ≥60 minutes at 2 degrees, lower bleaches warrant consideration.

Regression analyses revealed that subretinal drusenoid deposits were the strongest independent determinant of delayed RIT, with prolongations of +18.5 to +26.3 minutes across eccentricities. These data extend previous findings linking SDD with impaired rod-mediated recovery.^21^^,^^49^^,^^50^ Interestingly, these models explained 71% to 79% of total RIT variance, despite of the absence of local features. This again is compatible with previous data suggesting that subretinal drusenoid deposits are associated with scotopic dysfunction in a macula-wide manner instead of localized dysfunction.^19^^,^^51^^–^^53^ The associations between RIT and age observed are consistent with prior reports as well (2.41 minutes per decade at 2 degrees, 1.97 minutes per decade at 4 degrees, and 1.62 minutes per decade at 6 degrees). Previous studies have generally estimated age-related increases in RIT, or in cone–rod break time (a closely related measure in healthy participants), in the range of approximately 0.6 to 1.2 minutes per decade.^35^^,^^54^ However, those estimates were derived primarily from testing at more eccentric retinal locations, typically between 5 degrees and 12 degrees, which accounts for the somewhat smaller age slopes reported in those investigations.

### Limitations

Several limitations of the present study warrant consideration. The cohort was cross-sectional, precluding assessment of longitudinal change within individuals.

A limitation of the present study is that the age distribution differed substantially between healthy volunteers and patients with AMD. All analyses – including covariate-adjusted ROC analyses and the estimation of visual function parameter deviations for individual patients – rely on modeling the behavior of dark adaptation curve parameters in healthy individuals as a function of age. Whereas this approach allows estimation of expected age-related changes, it introduces assumptions. These assumptions may not fully capture the true relationship if, for example, visual function declines more steeply beyond a certain age or if interindividual or test–retest variability increases in older individuals. An age-matched control group would therefore be preferable, as it would allow direct comparison without reliance on modeling assumptions.

Healthy volunteers were selected based on OCT imaging demonstrating absence of drusen or other retinal abnormalities suggestive of retinal disease; however, formal AMD grading using the Beckman classification based on fundus photography was not performed in this group. Consequently, the presence of very early or subclinical AMD cannot be completely excluded. In the AMD cohort, disease severity was classified on a per-eye basis to allow evaluation of fellow-eye effects. This design resulted in a somewhat more advanced cohort than a strictly bilateral intermediate AMD population, as six participants (22.9%) had late AMD in the contralateral eye. Finally, although individuals with systemic conditions known to affect vitamin A metabolism (e.g. liver disease or malabsorption syndromes) were excluded, serum vitamin A levels were not measured, and unrecognized biochemical variability therefore cannot be ruled out.

Lens status was not formally accounted for in the analyses. In older subjects, cataract can attenuate retinal illuminance and thereby reduce effective bleach strength, whereas cataract extraction with intraocular lens implantation may alter retinal illumination characteristics and influence subsequent RIT measurements.^46^^,^^55^ These factors were not systematically evaluated. In addition, bleach strength was not adjusted for interindividual differences in pharmacologically dilated pupil size, which could introduce further variability in the measured responses. Last, our cross-sectional data imply parafoveal (2 degrees) dark adaptation measurements are prone to show greater change over time compared with more eccentric measurements. But longitudinal data are required to demonstrate this hypothesized pattern of functional disease progression. Despite the excellent explanatory power of the structure-function model based on global features, inclusion of other features – including refined severity scales,^56^ hyper-reflective foci,^57^ choriocapillaris flow deficits,^58^ and point by point comparison to retinal layer thicknesses^59^^,^^60^ – warrants considerations.

Fundus-tracked dark adaptometry provides a spatially precise assessment of rod-mediated dysfunction in AMD. Parafoveal RIT, particularly at 2 degrees, identifies the earliest and most consistent abnormality, marking the transition from delayed rod recovery to steady-state sensitivity loss. These results suggest that parafoveal dark adaptometry captures disease expression at a stage when the retinoid cycle is impaired, but photoreceptor structure remains largely intact, even in eyes with severe RIT delay. Although further longitudinal data are needed, RIT at 2 degrees eccentricity offers a mechanism-linked, quantifiable functional metric with potential value for monitoring early AMD progression and therapeutic response.

## Supplementary Material

Supplement 1
