# Supplementary material for: Parafoveal Dark Adaptation in Early and Intermediate Age-Related Macular Degeneration
Source: Invest Ophthalmol Vis Sci. 2026 May 29;67(5):74. doi: 10.1167/iovs.67.5.74 (PMC13225299; doi:10.1167/iovs.67.5.74)

SUPPLEMENT

Supplementary Table S1. Priors for the Bayesian Nonlinear Regression

Parameter	Unit	Priors for S-MAIA data
Initial threshold (t_0)	logUnits	Normal distribution (μ=2.5, SD=1)
Exponential cone recovery time constant (τ)	min	Exponential (λ=0.1)
Cone threshold (c_t)	logUnits	Normal distribution (μ=-0.7, SD=2)
Cone-rod break time (CRB)	min	Log-normal distribution (μ=4, σ=1)
S2 slope (S_2)	logUnits per min	Normal distribution (μ=-0.24, SD=0.05)
Final rod threshold (t_f)	logUnits	Normal distribution (μ=-2.5, SD=1.5)

Supplementary Table S2. Cohort Characteristics within AMD-Patients by Presence of Subretinal Drusenoid Deposits (SDD)

	SDD Absent (N=25)	SDD Present (N=10)
**Age [years]**		
Median [IQR]	69.1 [61.5, 75.3]	80.3 [71.3, 83.0]
**Sex**		
female	16 (64%)	6 (60%)
male	9 (36%)	4 (40%)
**Study eye**		
Left	8 (32%)	6 (60%)
Right	17 (68%)	4 (40%)
**Study eye visual acuity [logMAR]**		
Median [IQR]	-0.07 [-0.07, 0.1]	0.02 [-0.07, 0.17]
**Diagnosis: Study eye**		
eAMD	8 (32%)	3 (30%)
iAMD	16 (64%)	4 (40%)
late AMD: GA	1 (4%)	3 (30%)
**Diagnosis: Fellow eye**		
eAMD	8 (32%)	2 (20%)
iAMD	14 (56%)	3 (30%)
late AMD: GA	3 (12%)	3 (30%)
Late AMD: nAMD	0 (0%)	2 (20%)

eAMD = early AMD, iAMD = intermediate AMD, nAMD = neovascular AMD, GA = geographic atrophy

Supplementary Table S3. Dark Adaptometry Parameters within AMD Patients by Presence of Subretinal Drusenoid Deposits (SDD)

	SDD Absent (N=25)	SDD Present (N=10)
**Cone threshold at 2° [logUnits]**		
Median [IQR]	-0.58 [-0.68, -0.43]	-0.41 [-0.58, -0.36]
**Final (rod) threshold at 2° [logUnits]**		
Median [IQR]	-1.65 [-2.05, -0.95]	-0.41 [-0.58, -0.36]
**Rod intercept time (RIT) at 2° [min]**		
Median [IQR]	33.1 [22.7, 60.0]	60.0 [60.0, 60.0]
**Cone threshold at 4° [logUnits]**		
Median [IQR]	-0.57 [-0.66, -0.37]	-0.35 [-0.53, -0.15]
**Final (rod) threshold at 4° [logUnits]**		
Median [IQR]	-2.14 [-2.35, -1.81]	-0.54 [-1.30, -0.25]
**Rod intercept time (RIT) at 4° [min]**		
Median [IQR]	24.4 [15.6, 41.8]	60.0 [60.0, 60.0]
**Cone Threshold at 6° [logUnits]**		
Median [IQR]	-0.49 [-0.57, -0.35]	-0.32 [-0.36, -0.30]
**Final (rod) threshold at 6° [logUnits]**		
Median [IQR]	-2.33 [-2.45, -2.08]	-0.87 [-1.99, -0.13]
**Rod intercept time (RIT) at 6° [min]**		
Median [IQR]	21.2 [12.9, 38.6]	60.0 [45.7, 60.0]

Supplementary Table S4. Prevalence of Dark-Adaptation Abnormalities by Eccentricity: Evidence of a Parafoveal Gradient in AMD

Abnormal cone threshold (CT), final rod threshold (FT), and rod intercept time (RIT) were reported as rates outside age-adjusted normal limits.

N	Position	Abnormal CT [rate]	Abnormal FT [rate]	Abnormal RIT [rate]
35	2°	0.17	0.51	0.86
35	4°	0.20	0.37	0.69
35	6°	0.26	0.29	0.60

Supplementary Figure S1. Example of Fundus-controlled Two-color Dark Adaptation at 4°

The study participant was a female 72-year-old with intermediate AMD with a BCVA of -0.07 logMAR and no subepithelial drusenoid deposits. Sky-blue lines represent fitted model curves. Cone-rod break (CRB) is shown by the dashed line, rod intercept time (RIT) was defined as the time to detect a criterion stimulus of -1.4 logUnits, S2-slope (S_2) is represented by the red line, cone threshold (c_t) and final (rod) threshold (t_f) are marked on the right.

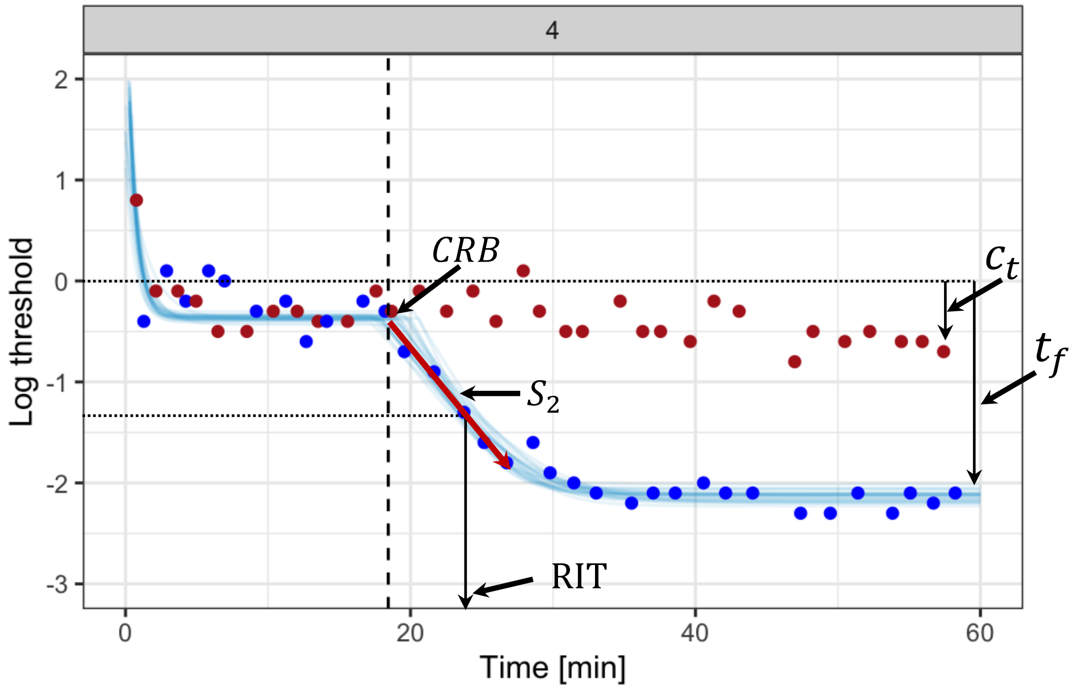

Supplement: Supplement 1 [file iovs-67-5-74_s001.pdf]
